# The Centennial Anniversary of the Massachusetts Medical Society

**Published:** 1881-09

**Authors:** 


### THE CENTENNIAL ANNIVERSARY OF THE MASSACHUSETTS MEDICAL SOCIETY.

Held at Boston, June 8th and 9th, 1881.

On the morning of June 8th, nearly eight hundred of the thirteen hundred and fifty members of the society had arrived at Boston, prepared to do their duty by the venerable organization of which they were ornaments. A special train, which was in waiting, quickly took such members as desired to Brighton, to view the slaughtering establishments, euphemistically known as abattoirs, there. These interesting places were thoughtfully inspected by the visitors. From Brighton the guests went to Cambridge, and the wonders of Harvard College and accessories were examined.

At 11 A.M. the Society assembled at Sander's Theatre, and was called to order by the President, Dr. Henry W. Williams. An address was then delivered by Dr. Samuel A. Green, on

THE HISTORY OF THE MASSACHUSETTS MEDICAL SOCIETY.

The speaker, after some preliminary remarks, said that the Massachusetts Medical Society was incorporated on November 1st, 1781, and its charter was signed by Samuel Adams, as President of the Senate, and John Hancock, as Governor of the Commonwealth.

There had been before this time a medical Society in Boston, which was the first one formed in America. It appears to have been in existence as early as the year 1735, though it did not continue long. Its records are irretrievably lost, and all that is known about it is gathered from fragmentary sources. It is very likely that it included in its list of members some of the ministers, as they were interested in the study and practice of medicine.

A long generation passed, and the Massachusetts Medical Society took the field, and occupied the broad limits of the State, including the District of Maine. Its members, together with the clergy, represented the education and refinement of the community. The charter members were thirty-one in number, and represented different sections of the State, fourteen of them belonging to Boston. The first meeting of the corporation was duly held in the County Court House, on November 28, 1781, at which time there were present nineteen of the thirty-one members. One of the prime objects of the society was to draw the line between the intelligent and ignorant practitioners of medicine. At the third meeting permanent officers were chosen, and Dr. Edward A. Holyoke was elected president. Dr. Holyoke practiced medicine in Salem for seventy-nine years, and it was said of him that there was not a dwelling house in the town at which he had not visited professionally. He died March 31, 1829, having reached the advanced age of more than one hundred years.

The speaker went on to give the history of the successive annual meetings during the first twenty years. He referred also to the establishment of the Harvard Medical School and the part which the society had in its growth and development.

At first, he said, the school was looked upon by the society with some jealousy, as it feared that the existence of two institutions would lead to serious embarrassment. Such feeling did not last long, however. At this time there were but three professors in the medical school, and two of these were original members of the medical society. It was, therefore, extremely improbable that there would be any permanent friction between the two bodies. The medical society had no right to confer degrees, and it does not appear that the medical school had any intention of granting testimonial letters to the profession at large.

It is a curious fact that the Chair of Anatomy in that school has had but three occupants in the ninety-eight years of its existence. These are Dr. John Warren, Dr. John Collins Warren and Dr. Oliver Wendell Holmes.

The history of the establishment of the Massachusetts General Hospital, in 1810, was related, and the active part taken by members of the society in introducing inoculation, and later, vaccination, was refered to as being most creditable to the intelligence and far-sightedness of the organization.

Regarding the comparative age of the society, Dr. Green said: The Massachusetts Medical Society is now the oldest State organization of a similar character in the country. Since its formation it has borne on its rolls the names of three thousand seven hundred persons, and today its membership includes one thousand three hundred and fifty physicians from all parts of the commonwealth. These members represent every section of the State, and their influence on one another is as immense as it is incalculable. The average attendance at the annual meetings of late years is not far from seven hundred and fifty members; these meetings last through two days, and, with few exceptions have been held in Boston. The charter of the New Jersy Medical Society antedates that of this society by some years, but there have been breaks in its regular line of descent.

After the address the members were invited by President Eliot to visit Harvard College, and a lunch was given in Memorial Hall. After this a train took them to Boston. An excursion down the bay and to Nantasket Beach occupied the rest of the afternoon.

In the evening a reception was tendered the society by Dr. Williams, which was much enjoyed. A number of physicians from out of the State were present, including, we are told by the local press, the venerable "Dr. Groce" and "Dr. Jacobie" (how narrow a thing is medical fame!).

*Second Day.*—The morning was occupied in part with more sight-seeing. The hospitals of the city and various museums were thrown open to the members, and were extensively visited by them. A special exhibition had been prepared and was displayed in Horticultural Hall. It consisted of a comparative and historical exhibition of botanic plates, of old books with quaint illustrations, of surgical instruments and other curious and useful objects belonging to the past and present times. On one side were the drugs in use more than one hundred years ago, and which still retained their ground; drugs introduced within the past century—a large collection; discarded remedies, drugs, and chemicals; new remedies not officinal—about fifty. A very extensive and complete collection of surgical instruments and appliances occupied the centre of the hall. Nearly two hundred botanical plates from the botanical gardens, Cambridge, adorned the walls, and a noticeable feature of the exhibition was the botanical models, made in Paris, from the College of Pharmacy. A rare department of the display was a large collection of ancient medical works, illustrated, some of them over three hundred years old; also anatomical and surgical charts.

The one hundredth regular annual meeting took place at 11 A. M., with the President, Dr. Williams in the chair. Not very much business was done.

By vote of the society, Dr. Calvin S. May, late Superintendent of the Danvers Insane Asylum, was expelled, because of his failure to answer satisfactorily charges of unprofessional conduct while in charge of that institution.

It was reported that eighty-two fellows had been added to the society during the past year, and that thirty had died. On motion of Dr. B. Joy Jeffreys, the following resolution was adopted:

WHEREAS, A petition has been presented to Congress asking for the calling of an international commission to consider and agree upon standard methods of testing visual acuteness and color blindness, and standard requirements of these necessary qualifications in the navies and merchant marines;

VOTED, that the Massachusetts Medidal Society heartily approves of the proposed international commission, and hereby directs the secretary of the society to transmit the vote to Congress when next assembled.

Dr. J. Collins Warren then delivered the annual address. He began with a sketch of the history of the society, and referred to the many beneficent results which had been secured by its agency; the introduction of vaccination, the establishment of the Massachusetts General Hospital, the abolishment of the coroners' system, etc. The speaker then referred to the subject of medical societies in general, and how they should be organized and managed, so as to accomplish the most good, He criticised the American Medical Association, and illustrated by it and certain State societies how comparatively inefficient medical organizations may be.

Dr. Warren then touched upon a number of other subjects which, he thought, properly deserve earnest attention from physicians and medical societies. These were the establishment of State boards of health, of which there are now twenty-nine; the regulation of the practice of medicine, and the subject of the medical education of women. The speaker urged a change in the laws relating to expert medical testimony, so that there might be an efficient method and men selected who really represented the highest training of the profession. He referred to the subject of medical journalism. He thought that the days of aunual and quarterly publications were passing away. Societies ought to arrange to publish their proceedings at frequent intervals, in order to make their work more valuable. Other suggestions were made as to the best way in which society work should be done.

The speaker closed with mention of the responsibility falling on the young members, and with hope for their future.

In the afternoon a grand banquet was served in Music Hall. A thousand plates were laid for the guests. Dr. J. C. White presided, with Governor Long on one side and President Williams on the other.

After the dinner many toasts were given and speeches made. With some opening remarks, Dr. White introduced Dr. Williams, who made in response to a toast from Dr. Williard Parker: "The Massachusetts Medical Society: as years roll on may she ever unite with the wisdom and experience of age the vigor and efficiency of youth."

Dr. Williams was followed by Governor Long, President Elliot, and Judge Hoar.

The last speaker told of his early prejudice against the doctors, manifested during an attack of measels at four years of age, but said that his respect for them had steadily increased since. To quote from Scripture, "as certain of your poets have said:"

> "Little of all we value here
> Wakes on the morn of its hundredth year
> Without both feeling and looking queer.
> In fact, there's nothing that keeps its youth,
> So far as I know, but a tree and truth."

As the Judge said this he turned in acknowledgement to Dr. Holmes, who sat near him, and the hand-clapping was cordial in compliment to the Professor. Then turning the quotation from the "One-Hoss Shay" to the society, Judge Hoar eulogized its youth, its strength, and its efficiency. Then he mentioned the growing freedom from personal and professional bickerings, the elevation of professional knowledge, the progress in discovery, "the substitution of Apollo for Mercury as their patron deity," and the abolition of the ancient pretence which was once the curse of the profession. Now the profession does not rely so much as once it did upon the art of seeming wise.

Dr. Holmes read a poem, which was warmly applauded. Its publication is withheld for the present.

Dr. Holmes was followed by Rev. Phillips Brooks, Dr. S. D. Gross, Rev. Dr. Ellis, and Professor Alexander Agassiz.

The dinner concluded the celebration, and the guests departed, all apparently having had a most enjoyable time.—*N. Y. Medical Record.*

